# Supplementary material for: Infertility screening in unmarried women: A scoping review protocol
Source: PLoS One. 2025 Aug 14;20(8):e0329899. doi: 10.1371/journal.pone.0329899 (PMC12352679; doi:10.1371/journal.pone.0329899)
Supplement: S1 File — (DOCX) [file pone.0329899.s002.docx]

**S2 File. Search strategy** (Terms can be used in the search strategy in PubMed database).

| Number | Search terms | | |
| --- | --- | --- | --- |
|  | Concept | | Syntax |
| #1 | **P** | **Participants** | Unmarried women OR Unmarried woman OR single Women OR single Woman OR Never Married Women OR Never Married Woman |
| #2 | **C** | **Concept** | Infertility Screening[Title/Abstract] OR Infertility evaluation[Title/Abstract] OR Infertility diagnosis[Title/Abstract] OR Fertility testing[Title/Abstract] OR Fertility assessment[Title/Abstract] OR reproductive health screening[Title/Abstract] OR Reproductive function testing[Title/Abstract] OR sterility assessment[Title/Abstract] OR conception evaluation[Title/Abstract] OR infertility diagnostics[Title/Abstract] OR Subfertility screening[Title/Abstract] OR sub-fertility screening[Title/Abstract] OR bareness[Title/Abstract] OR infecundity[Title/Abstract] |
| #3 | #1 AND #2 | | |
| #4 | from inception to May 31, 2025 | | |
| #5 | #3 AND #4 | | |
