# Supplementary material for: Infertility screening in unmarried women: A scoping review protocol
Source: PLoS One. 2025 Aug 14;20(8):e0329899. doi: 10.1371/journal.pone.0329899 (PMC12352679; doi:10.1371/journal.pone.0329899)
Supplement: Attachment — Submitted filename: Infertility Screening in Unmarried Women.docx [file pone.0329899.s003.docx]

I**nfertility Screening in Unmarried Women: A Scoping Review Protocol - PONE-D-25-15839**

This is an interesting area and the core elements of a review protocol have been included. However I have a few comments for the authors.

- What is your definition of unmarried women? Is it never been married? Is it those who have been married before and are currently divorced/single? Is it those who are cohabiting? As this might have a bearing on your articles
- Why are you restricting to 1990-2025 period? You might need to justify
- Line 20 is the Lead author part of the independent extractors?
- Line 33 – You might need to write ISI in full for the first time
- I think you should also mention the use of wild cards and truncations to broaden the database search
- Are you also going to including the 6^th^ optional process by Arksey and O’Malley of consultative exercise as this might provide useful information if key stakeholders are engaged in the finalization of search strategy and grey literature identification
- May you consider provision of an example of the search strategy key words table
- Take note that some words such as sub-fertility, bareness, sterility and infecundity might need to be included in the search strategy
- Who will do the search of studies? Will it be done by one person the lead author or by the independent reviewers? Will this be done separately? Are you involving any expert in Library and Information retrieval to assist with the searching?
- Line 135 Who is S.B? Is this the lead author being referred to? Who will contact him/her?
- Line 141 Joanna Briggs (JBI) have developed critical appraisal tools which you might consider to use
- Line 144 You might need to include thematic analysis for the qualitative and mixed methods studies
- Line 154 I would propose that you rephrase that since quality assessment is optional, the authors will not consider doing that process.
- Line 156 This is debatable on the usefulness of the finding for policy if the quality of studies are not assessed/ critically appraised. You might need to consider revising to include critical appraisal
- References – You might need to consider citing articles that are more current e.g 5 years since there is quite a number of literature on infertility. The methodological citations are ok.
