# Supplementary material for: Infertility screening in unmarried women: A scoping review protocol
Source: PLoS One. 2025 Aug 14;20(8):e0329899. doi: 10.1371/journal.pone.0329899 (PMC12352679; doi:10.1371/journal.pone.0329899)
Supplement: Attachment — Submitted filename: Response to Reviewers 2025 06 06.docx [file pone.0329899.s005.docx]

Dear Dr. Godwin Banafo Akrong, 2025/06/06

Academic Editor of PLOS ONE

Thank you for your email and the opportunity to revise our manuscript. We appreciate the time and insights of the reviewers and believe that their comments have helped us to significantly improve the manuscript. We have carefully considered all of the comments and have revised the manuscript accordingly.

Below, we provide a point-by-point response to each of the editor and reviewers' comments.

I encourage you to address all the reviewers' (Reviewer #1 and #2) comments and make the necessary revisions, particularly in improving the PRISMA flow diagram and the critical discussion of gaps and methods sections. I look forward to reviewing your revised manuscript.

**Response:** We acknowledge the importance of addressing all the comments provided by both Reviewer #1 and Reviewer #2. We are committed to carefully considering each point and making the necessary revisions to improve the quality and clarity of our protocol.

**Journal Requirements:**

Comment 1. Please ensure that your manuscript meets PLOS ONE's style requirements, including those for file naming. The PLOS ONE style templates can be found at <https://journals.plos.org/plosone/s/file?id=wjVg/PLOSOne_formatting_sample_main_body.pdf> and <https://journals.plos.org/plosone/s/file?id=ba62/PLOSOne_formatting_sample_title_authors_affiliations.pdf>.

**Response:** Thank you for this important point. We have carefully reviewed the PLOS ONE style guidelines and made the necessary revisions to ensure compliance. As these revisions primarily involved formatting adjustments, such as file naming and layout, we have not highlighted them. However, we confirm that all changes have been made according to the journal's requirements.

Comment 2. In the online submission form you indicate that your data is not available for proprietary reasons and have provided a contact point for accessing this data. Please note that your current contact point is a co-author on this manuscript. According to our Data Policy, the contact point must not be an author on the manuscript and must be an institutional contact, ideally not an individual. Please revise your data statement to a non-author institutional point of contact, such as a data access or ethics committee, and send this to us via return email. Please also include contact information for the third party organization, and please include the full citation of where the data can be found.

**Response:** No datasets were generated or analysed during the current study. All relevant data from this study will be made available upon study completion. The sentence was corrected.

Comment 3. Please remove your figures from within your manuscript file, leaving only the individual TIFF/EPS image files, uploaded separately. These will be automatically included in the reviewers’ PDF.

**Response:** The figures will be removed from the manuscript file, and the individual TIFF image file uploaded separately.

**Reviewer #1:**

This is an interesting area and the core elements of a review protocol have been included. However I have a few comments for the authors.

Comment 1. What is your definition of unmarried women? Is it never been married? Is it those who have been married before and are currently divorced/single? Is it those who are cohabiting? As this might have a bearing on your articles

**Response:** For the purpose of this scoping review, "unmarried women" refers to women who have never been married. (Page 5, Line 92)

Comment 2. Why are you restricting to 1990-2025 period? You might need to justify

**Response:** The search covers publications from inception to May 31, 2025. (Page 5, Lines 95)

Comment 3. Line 20 is the Lead author part of the independent extractors?

**Response:** Currently, the two authors listed are involved in the initial stages of the protocol development. Additional collaborators will join the team for the full review process, and their specific roles, including their involvement in independent data extraction, will be clearly defined and acknowledged in the final manuscript.

Comment 4. Line 33 – You might need to write ISI in full for the first time.

**Response:** "ISI" be written out in full as "Institute for Scientific Information" upon its first mention in the revised manuscript. (Page 6, Line 110)

Comment 5. I think you should also mention the use of wild cards and truncations to broaden the database search

**Response:** Thank you for the suggestion. We include a statement in the search strategy section specifying the use of wildcards and truncation to ensure a broader and more comprehensive search across the databases. (Page 6, Lines 108-109)

Comment 6. Are you also going to including the 6th optional process by Arksey and O’Malley of consultative exercise as this might provide useful information if key stakeholders are engaged in the finalization of search strategy and grey literature identification

**Response:** The consultative exercise, as suggested by Arksey and O'Malley, is a valuable approach. While not explicitly listed as a separate step in our 14-step framework which is based on their methodology and incorporates improvements by Tricco and Peters, the principles of engaging with key stakeholders will be integrated into the literature search and grey literature identification stages to ensure a comprehensive and relevant review. (Page 5, Lines 101-103)

Comment 7. May you consider provision of an example of the search strategy key words table

**Response:** We appreciate this valuable suggestion. We have added an S2 File illustrating our search strategy keyword. (Page 5, Line 111)

Comment 8. Take note that some words such as sub-fertility, bareness, sterility and infecundity might need to be included in the search strategy

**Response:** We appreciate this valuable feedback. We've reviewed our search strategy and have incorporated the suggested keywords. (Page 5, Line 111)

Comment 9. Who will do the search of studies? Will it be done by one person the lead author or by the independent reviewers? Will this be done separately? Are you involving any expert in Library and Information retrieval to assist with the searching?

**Response:** We appreciate your questions regarding our search strategy. Initial search for studies will be conducted by S.B.. We won't be seeking assistance from an external expert in Library and Information retrieval for the search itself. However, to ensure the robustness of our search strategy, we will consult with subject-matter experts in the field when developing and refining the keywords used. (Page 6, Lines 114-117)

Comment 10. Line 135 Who is S.B? Is this the lead author being referred to? Who will contact him/her?

**Response:** Thanks for pointing this out. We've clarified in the manuscript that S.B. refers to the principal investigator. S.B. will be contacted for any essential missing information to ensure a complete data synthesis. (Page 6, Line 114)

Comment 11. Line 141 Joanna Briggs (JBI) have developed critical appraisal tools which you might consider to use

**Response:** We appreciate the suggestion regarding Joanna Briggs Institute (JBI) critical appraisal tools. However, consistent with scoping review methodology, we will not be conducting a formal quality appraisal of the included studies. This approach aligns with established guidelines for scoping reviews, as cited in reference (1).

Comment 12. Line 144 You might need to include thematic analysis for the qualitative and mixed methods studies

**Response:** Thank you for this excellent point. We agree that thematic analysis is crucial for qualitative and mixed-methods studies. We've clarified our Data Synthesis and Analysis section to explicitly state that thematic analysis will be employed for qualitative data, in addition to the tabular and diagrammatic synthesis and narrative summary already mentioned. (Page 8, Lines 153-154)

Comment 13. Line 154 I would propose that you rephrase that since quality assessment is optional, the authors will not consider doing that process.

**Response:** Thank you for this crucial feedback. We removed this sentence.

Comment 14. Line 156 This is debatable on the usefulness of the finding for policy if the quality of studies are not assessed/ critically appraised. You might need to consider revising to include critical appraisal

**Response:** We appreciate the reviewer's point regarding the importance of critical appraisal. While we acknowledge its value, a formal quality assessment of individual studies was beyond the scope of this foundational study, which aims to provide an initial critical foundation.

Comment 15. References – You might need to consider citing articles that are more current e.g 5 years since there is quite a number of literature on infertility. The methodological citations are ok.

**Response:** We thank the reviewer for their valuable feedback on the currency of the references. While we appreciate the suggestion to limit citations to the last five years to reflect the most recent literature in the rapidly evolving field of infertility, we believe that a 10-year timeframe is more appropriate for this manuscript. This extended period allows for the inclusion of foundational studies and established methodological papers that provide essential context and are still widely accepted. Our approach was to balance the inclusion of seminal works with current findings to offer a comprehensive overview. We have, however, re-examined our references to ensure that the most critical recent literature is well-represented while retaining the key publications from the last decade that are fundamental to the arguments presented.

**Reviewer #2:**

Thank you for submitting your paper on this important and timely topic. Below are my suggestions to strengthen the manuscript:

**Critical Discussion of Gaps and Methods:**

Comment 1. It would be valuable to include a critical discussion of the identified gaps in male infertility screening (e.g., limitations in current guidelines, underrepresented populations, or unmet needs in screening protocols).

**Response:** Thank you for your valuable feedback. We note that the comment refers to male infertility screening, whereas our study focuses on infertility screening in unmarried women. In the final manuscript, we will include a critical discussion. (Page 4, Line 71)

Comment 2. Additionally, a summary of the methods currently used for infertility screening would provide useful context for readers.

**Response:** Thank you for the suggestion. We will include a concise summary of current infertility screening methods to provide better context for readers. (Page 3, Lines 57-62)

**PRISMA Flow Diagram:**

Comment 3. The PRISMA diagram is currently empty, please confirm whether this is an upload error. If not, the absence of this data raises concerns about the study’s findings, as the screening process is a cornerstone of a scoping review.

**Response:** Thank you for your feedback. We confirm the PRISMA diagram was inadvertently omitted due to an upload error. (Page 7, Line 128)

Comment 4. I recommend moving the PRISMA diagram from the appendices to the Methods section to improve readability and allow readers to follow the screening process seamlessly. Accompanying the diagram with a brief narrative (e.g., number of records screened, excluded, and reasons for exclusion) would further enhance clarity.

**Response:** Thank you for the suggestion. We will move the PRISMA diagram to the Methods section. (Page 7, Line 128)

**Methods Section Clarity and Completeness:**

Comment 5. Consider relocating other relevant tables (e.g., search strategies, data extraction frameworks) from the appendices to the main Methods section

**Response:** Thank you for the suggestion. We believe retaining tables like search strategies and data extraction frameworks as Supporting Information is more appropriate, as it keeps the main Methods section concise while ensuring detailed information remains accessible for interested readers.

While revising your submission, please upload your figure files to the Preflight Analysis and Conversion Engine (PACE) digital diagnostic tool, [https://pacev2.apexcovantage.com/](https://track.editorialmanager.com/CL0/https:%2F%2Fpacev2.apexcovantage.com%2F/1/010f019595c7946a-d7948cc1-9fdf-439c-8b44-e645c1f57b77-000000/GfdVlmzbVdNsNpJAAcKcdoxNqDnHRCpiiz-0WQbuNJQ=202). PACE helps ensure that figures meet PLOS requirements. To use PACE, you must first register as a user. Registration is free. Then, login and navigate to the UPLOAD tab, where you will find detailed instructions on how to use the tool. If you encounter any issues or have any questions when using PACE, please email PLOS at [figures@plos.org](mailto:figures@plos.org). Please note that Supporting Information files do not need this step.

**Response:** We add a figure in this manuscript with requirements checking.

**References:**

1. Tricco AC, Lillie E, Zarin W, O'Brien KK, Colquhoun H, Levac D, et al. PRISMA Extension for Scoping Reviews (PRISMA-ScR): Checklist and Explanation. Annals of Internal Medicine. 2018;169(7):467-73.
