# Supplementary material for: Infertility screening in unmarried women: A scoping review protocol
Source: PLoS One. 2025 Aug 14;20(8):e0329899. doi: 10.1371/journal.pone.0329899 (PMC12352679; doi:10.1371/journal.pone.0329899)
Supplement: Attachment — Submitted filename: Response to Reviewers 2025 06 25.docx [file pone.0329899.s006.docx]

Dear Dr. Godwin Banafo Akrong, 2025/06/25

Academic Editor of PLOS ONE

Thank you for your email and the opportunity to revise our manuscript. We appreciate the time and insights of the reviewers and believe that their comments have helped us to significantly improve the manuscript. We have carefully considered all of the comments and have revised the manuscript accordingly.

Below, we provide a point-by-point response to each of the editor and reviewers' comments.

**ACADEMIC EDITOR:**

I encourage you to address all the concerns raised by Reviewer #2 and make the necessary revisions. I look forward to reviewing your revised manuscript.

**Response:** We appreciate your feedback and the opportunity to revise our manuscript. We will carefully address all the concerns raised by Reviewer #2 and make the necessary revisions to the manuscript. We are committed to submitting a revised version that fully addresses these points.

**Journal Requirements:**

Comment 1. Please review your reference list to ensure that it is complete and correct. If you have cited papers that have been retracted, please include the rationale for doing so in the manuscript text, or remove these references and replace them with relevant current references. Any changes to the reference list should be mentioned in the rebuttal letter that accompanies your revised manuscript. If you need to cite a retracted article, indicate the article’s retracted status in the References list and also include a citation and full reference for the retraction notice.

**Response:** We have thoroughly reviewed our reference list to ensure its completeness and accuracy. We confirm that none of the cited papers have been retracted. All references are current and relevant to our manuscript.

**Reviewer #1:**

The authors have throughly addressed the comments raised in the initial review and I recommend for submission

**Response:** We sincerely thank Reviewer #1 for their positive feedback.

**Reviewer #2:**

Comment 1. The abstract is poorly written. The authors should please follow the criteria presented by the journal when it comes to drafting an abstract. Kindly merge the sections presented into one paragraph.

**Response:** Thanks for your feedback on the abstract's structure. While we appreciate the suggestion to merge it into a single paragraph, we've intentionally kept the current segmented format with distinct headings. As a scoping review protocol, this detailed, sectioned approach significantly enhances clarity and navigability. It helps readers and reviewers quickly understand specific planned steps. We believe this structure best serves the purpose of a protocol, optimizing its utility. We also note that the journal guidelines do not mandate a single-paragraph format for the abstract. We've ensured the abstract adheres to all other journal guidelines, such as word count and exclusion of citations.

Comment 2. The inclusion and exclusion criteria should be moved to the method section of their paper.

**Response:** Thank you for your valuable comment. For a scoping review protocol, we've intentionally kept "Inclusion and exclusion criteria" as a distinct section, separate from "Methods." In a protocol, these criteria are foundational; they define the review's scope immediately after the research questions. This upfront placement enhances clarity and transparency, helping readers quickly understand the review's boundaries before delving into the specific methodological steps. While they inform the methods, they are crucial, standalone components for a protocol.

Comment 3. The author should kindly pay attention to the tense in which they report their study since this work has already been carried out. This concern runs throughout the entire work. Example: “We won't be seeking assistance from an external expert in Library and Information retrieval for the search itself. However, to ensure the robustness of our search strategy, we will consult with subject-matter experts in the field when developing and refining the keywords used.”.

**Response:** Thank you for your valuable feedback regarding tense usage throughout the manuscript. We appreciate you highlighting this concern. However, we would like to clarify that this manuscript is a scoping review protocol, not a completed study. As such, it outlines the planned methodology and future actions for a review that is yet to be conducted. However we revise "Won't" to "will not".

While revising your submission, please upload your figure files to the Preflight Analysis and Conversion Engine (PACE) digital diagnostic tool, [https://pacev2.apexcovantage.com/](https://track.editorialmanager.com/CL0/https:%2F%2Fpacev2.apexcovantage.com%2F/1/010f019595c7946a-d7948cc1-9fdf-439c-8b44-e645c1f57b77-000000/GfdVlmzbVdNsNpJAAcKcdoxNqDnHRCpiiz-0WQbuNJQ=202). PACE helps ensure that figures meet PLOS requirements. To use PACE, you must first register as a user. Registration is free. Then, login and navigate to the UPLOAD tab, where you will find detailed instructions on how to use the tool. If you encounter any issues or have any questions when using PACE, please email PLOS at [figures@plos.org](mailto:figures@plos.org). Please note that Supporting Information files do not need this step.

**Response:** We add a figure in this manuscript with requirements checking.
